# Reduced tumor stiffness quantified by tomoelastography as a predicative marker for glypican-3-positive hepatocellular carcinoma

**DOI:** 10.3389/fonc.2022.962272

**Published:** 2022-11-28

**Authors:** Yihuan Wang, Jing Guo, Di Ma, Jiahao Zhou, Yuchen Yang, Yongjun Chen, Huafeng Wang, Ingolf Sack, Ruokun Li, Fuhua Yan

**Affiliations:** ^1^ Department of Radiology, Ruijin Hospital, Shanghai Jiao Tong University School of Medicine, Shanghai, China; ^2^ Department of Radiology, Charité–Universitätsmedizin Berlin, Berlin, Germany; ^3^ Department of General Surgery, Ruijin Hospital, Shanghai Jiao Tong University School of Medicine, Shanghai, China; ^4^ Department of Pathology, Ruijin Hospital, Shanghai Jiao Tong University School of Medicine, Shanghai, China

**Keywords:** magnetic resonance elastography (MRE), hepatocellular carcinoma (HCC), molecular target, extracellular matrix (ECM), biomechanics

## Abstract

**Background:**

Glypican-3 (GPC3) expression is investigated as a promising target for tumor-specific immunotherapy of hepatocellular carcinoma (HCC). This study aims to determine whether GPC3 alters the viscoelastic properties of HCC and whether tomoelastography, a multifrequency magnetic resonance elastography (MRE) technique, is sensitive to it.

**Methods:**

Ninety-five participants (mean age, 58 ± 1 years; 78 men and 17 women) with 100 pathologically confirmed HCC lesions were enrolled in this prospective study from July 2020 to August 2021. All patients underwent preoperative multiparametric MRI and tomoelastography. Tomoelastography provided shear wave speed (*c, m/s*) representing tissue stiffness and loss angle (*φ, rad*) relating to viscosity. Clinical, laboratory, and imaging parameters were compared between GPC3-positive and -negative groups. Univariable and multivariable logistic regression were performed to determine factors associated with GPC3-positive HCC. The diagnostic performance of combined biomarkers was established using logistic regression analysis. Area-under-the-curve (AUC) analysis was done to assess diagnostic performance in detecting GPC3-positive HCC.

**Findings:**

GPC3-positive HCCs (n=72) had reduced stiffness compared with GPC3-negative HCCs (n=23) while viscosity was not different (c: 2.34 ± 0.62 versus 2.72 ± 0.62 m/s, *P*=0.010, *φ*: 1.11 ± 0.21 vs 1.18 ± 0.27 rad, *P*=0.21). Logistic regression showed *c* and elevated serum alpha-fetoprotein (AFP) level above 20 ng/mL were independent factors for GPC3-positive HCC. Stiffness with a cutoff of *c* = 2.8 m/s in conjunction with an elevated AFP yielded a sensitivity of 80.3%, specificity of 70.8%, and AUC of 0.80.

**Interpretation:**

Reduced stiffness quantified by tomoelastography may be a mechanical signature of GPC3-positive HCC. Combining reduced tumor stiffness and elevated AFP level may provide potentially valuable biomarker for GPC3-targeted immunotherapy.

## Introduction

Hepatocellular carcinoma (HCC) ranks as the third leading cause of cancer death worldwide (1). Early-stage HCC can be treated curatively with surgical resection, transplantation, transarterial chemoembolization, or ablation. Despite therapeutic advances, less than 40% of HCC patients are eligible for potentially curative treatment (2).

Multityrosine kinase inhibitors, such as sorafenib, were the first systemic therapy for advanced HCC (3). However, due to a strong and broad resistance of HCC to cytotoxic chemotherapy, systemic therapy based on antiangiogenic tyrosine kinase inhibitors has been used primarily in advanced disease (4, 5). Recently, the quest for HCC treatment has focused on tumor antigen-specific immunotherapy and other approaches modulating the immunogenicity of HCC (6). Meanwhile, agents targeting the programmed cell death protein-1 and cytotoxic T lymphocyte antigen 4 have been approved for HCC treatment (7–9). Despite these encouraging developments in immunotherapy, there is an ongoing need to identify further molecular targets and develop biomarkers to assess treatment responses.

Glypican-3 (GPC3), a member of heparan sulfate proteoglycans anchored to the cell membrane, is highly expressed in >60% of all HCCs but not in benign hepatic lesions, hepatic cirrhosis, hepatitis, or in healthy liver tissue (10–12). Its overexpression has been associated with poorer prognosis (10, 13–15) and identified as a rational specific diagnostic biomarker or target for immunotherapy in HCC (16, 17). Various immunotherapies targeting GPC3 have been under investigation, including GPC3-targeted antibody treatment, peptide/DNA vaccine treatments, chimeric antigen receptor T cells therapy, immunotoxin use, and genetic therapies (10). he usefulness importance of GPC3 as a therapeutic target for both antibody- and cell-based immunotherapies has been explored in previous studies (18). Currently, GPC3 expression is detected mainly through immunohistochemical staining of HCC tissue samples obtained by surgical resection or fine-needle biopsy (10). Given the diagnostic and therapeutic importance of GPC3 and the lack of non-invasive detection methods for GPC3, a quantitative imaging biomarker for the detection of GPC3 with high sensitivity and specificity is urgently needed.

Tomoelastography, an advanced MR elastography (MRE) technique based on multiple frequencies, is an emerging noninvasive imaging technique for quantifying biomechanical properties of soft tissues *in vivo* (19). Tomoelastography yields quantitative maps of shear wave speed (*c* in m/s) and loss angle (*φ* in rad) as surrogates of tissue stiffness and viscosity, respectively. Tomoelastography has been applied for the biomechanical characterization of a variety of tumors *in vivo*, including pancreatic cancer (20, 21), neuro-tumors (22), prostate cancer (23, 24), rectal carcinoma (25), and liver tumors (26, 27). These studies have unveiled the relationship between biomechanical properties and changes in tissue microstructure associated with tumor progression including remodeling of the extracellular matrix (ECM) and collective cellular behavior (20, 26).

We hypothesize that biomechanical parameters might be sensitive to GPC3 expression in HCC as GPC3 is an ECM component that mediates cell-ECM and cell-cell interactions and promotes cell growth. To test this hypothesis, we conducted an exploratory study using tomoelastography to investigate the correlation between the biomechanical properties of HCC and their GPC3 expression levels, and to develop prediction models of GPC3-positive HCC.

## Material and methods

### Study population

**Figure 1:**
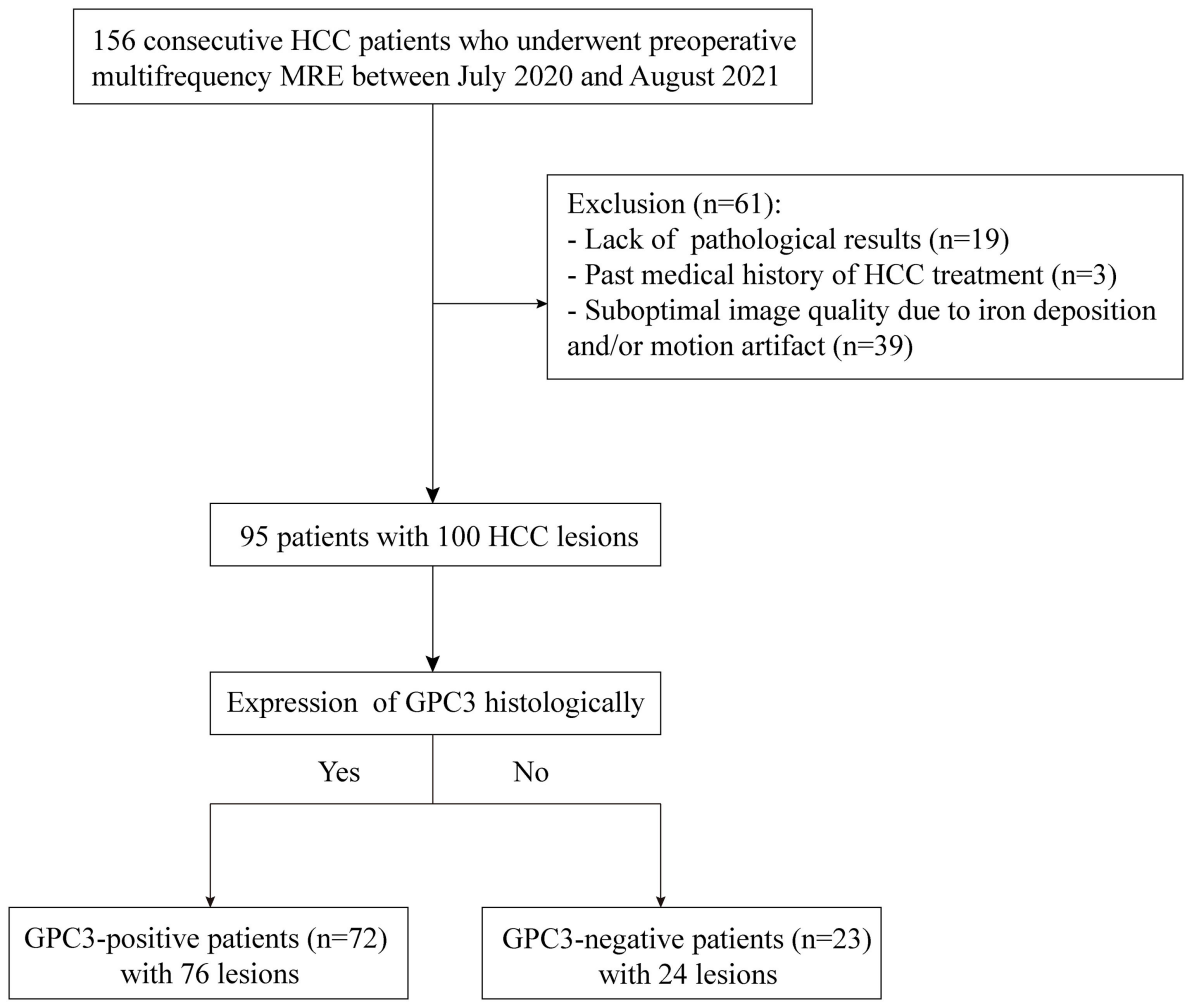
Flowchart of participant inclusion and exclusion.

This prospective single-center cohort study was approved by the institutional review board (No. RJ2018-209), and written informed consent was obtained from all study participants. From July 2020 to August 2021, 156 consecutive participants with suspected HCC were enrolled and underwent preoperative tomoelastography. Sixty-one participants were excluded due to lack of pathological results (n = 19), previous HCC treatment (n = 3), or poor MRI image quality due to iron deposition and/or motion artifacts (n = 39). Finally, 95 participants (mean age, 58 years ± 1; 78 men and 17 women) with 100 HCC lesions were included. A flowchart of participant recruitment is shown in **Figure 1**.

### Clinical MRI for HCC diagnosis

All participants underwent routine multiparametric MRI, which consisted of T1-weighted, T2w, diffusion-weighted-imaging with *b*-values of 0, 50, and 800 s/mm^2^, and multiphase dynamic contrast-enhanced imaging with Gd-DTPA. These routine clinical MRI examinations were performed within one week before surgery on systems from different vendors (Siemens, Philips, United Imaging) depending on their availability. All imaging parameters of the multiparametric MRI protocol are compiled in **Supplemental Table T1**.

### Tomoelastography

Tomoelastography examination was performed on a 1.5-Tesla MRI scanner (Magnetom Aera, Siemens, Erlangen, Germany) one day before surgery for all patients. The setup was similar to that described in Shahryari.et al (27). Briefly, mechanical vibration of 30, 40, 50, and 60 Hz were generated and transferred sequentially to the liver using four pressure pads driven by compressed air. Two anterior and two posterior pads, operating at 0.4 and 0.6 bar, respectively, were placed near the liver region. Three-dimensional wave fields were acquired using a single-shot, spin-echo echo-planar MRI sequence with flow-compensated motion-encoding gradients. Fifteen contiguous axial slices with a field of view of 384×312 mm^2^ (matrix size: 128×104) and 3×3×5 mm^3^ resolution were acquired during free breathing as proposed in Shahryari.et al (28). Further imaging parameters were: echo time of 59 ms; repetition time of 2050 ms; parallel imaging with GRAPPA factor 2; MEG frequency of 43.48Hz for 30, 40, 50Hz vibration frequencies and 44.88Hz for 60Hz vibration frequency; MEG amplitude of 30mT/m. Total acquisition time was approximately 3.5 mins. Multifrequency wavefield data were processed using the pipeline available at https://bioqic-apps.com. Full field-of-view maps of shear wave speed (*c*) and loss angle (*φ*) of the complex shear modulus were generated. As *c* is proportional to the square root of the storage modulus (real part of the complex shear modulus) while *φ* continuously changes from 0 (pure solid properties) to π/2 (pure fluid properties), these two parameters are also considered surrogates for stiffness and tissue fluidity, respectively. Henceforth, we will use *c* and *φ* for providing quantitative information, while “stiffness” and “fluidity” are reserved for discussing qualitative parameter changes.

### Image analysis

Based on imaging features such as tumor size, non-rim arterial phase enhancement (APHE), non-peripheral washout (“washout”) in the portal venous phase (PVP) or delayed phase (DP), and enhancing capsule (“capsule”), Liver Imaging Reporting and Data System (LI-RADS) categories ranging from LR1 to LR5 as well LR-M (malignant, not HCC-specific) and LR-TIV (Tumor In Vein) were assigned to each lesion using LI-RADS version 2018 (29).

For tomoelastography, regions of interest (ROIs) were manually drawn on T2w tomoelastography magnitude images using conventional T2w MR images for anatomical orientation. One main slice showing the primary lesion at its largest cross-sectional extension and its two adjacent slices were selected for defining ROIs of tumors. ROIs were also manually drawn to encompass as much of the background liver as possible on three consecutive sections with the largest liver cross-sectional coverage on the central *c*- and *φ*-map slices. The measurement results were averaged and then used as the representative parameters. Additionally, two radiologists - Rater#1 with 12 years of experience and Rater#2 with 2 years of experience – independently analyzed tomoelastography data in all 95 patients for testing interobserver variability.

### Histopathological analysis

Lesion specimens were obtained from surgical resection. Presence of microvascular invasion, Edmondson-Steiner grade, liver fibrosis stage, and inflammation grade were assessed in hematoxylin and eosin (H&E)-stained specimens. Immunochemistry staining was performed to verify GPC3 expression based on protocol described in Feng et al. (30). A sample was classified as positive for GPC3 expression when >10% of tumor cells showed GPC3 cytoplasmic staining. Ki-67 expression was assessed by noting the percentage of positively stained cells. All specimens were analyzed by a pathologist with 16 years of experience in hepatic pathology who was blinded to all radiological and clinical results.

### Statistical analysis

For group comparison, the χ2 test was used for qualitative parameters while Student′s t-test or the Mann-Whitney U-test was applied for quantitative measures. Interobserver agreement regarding mechanical parameters was tested using intraclass correlation coefficients (ICCs). Univariable and multivariable analyses of a backward logistic regression were used to determine predictive factors for GPC3-positive HCC. The diagnostic model was established using logistic regression analysis. Area-under-the-curve (AUC) analysis was done to assess diagnostic performance in detecting GPC3-positive HCC. AUC values were compared using the Delong test. All statistical analyses were performed with SPSS software (version 26; SPSS), GraphPad Prism software (GraphPad Prism for Windows, version 8.0), and MedCalc software (MedCalc Software Ltd). *P* < 0.05 was considered to indicate statistically significant differences.

## Results

### Clinicopathological characteristics of participants

The GPC3-positive group included 72 participants (mean age, 58 years ± 12; 57 men and 15 women) with 76 lesions while the GPC3-negative group included 23 participants (mean age, 60 years ± 9; 21 men and 2 women) with 24 lesions. In participants with multiple lesions, all lesions of the same individual were found to be either positive or negative for GPC3 expression.

Compared with the GPC3-negative group, GPC3-positive participants had elevated AFP levels (≥ 20 ng/ml, *P* < 0.001) and higher Ki-67-positive cellular index (Ki-67 ≥ 20%, *P* = 0.01). The two groups did not differ significantly in terms of demographic, laboratory, and pathologic parameters such as tumor size (*P* = 0.89), MVI (*P* = 0.90), Edmondson-Steiner grade (*P* = 0.08), liver fibrosis (*P* = 0.79), and inflammation grade (*P* = 0.33). The clinicopathological characteristics are summarized in **Table 1**.

**Table 1:** Comparison of clinicopathologic characteristics of participants with GPC3-positive and GPC3-negative HCC.

Characteristic	GPC3-positive group (n=72)	GPC3-negative group (n=23)	P Value
Participants			
Age (years) (range)	58 ± 12 (23-81)	60 ± 9 (41-75)	0.42
Sex (male: female)	57:15	21:2	0.31
BMI (kg/m^2^)	23.13 ± 3.32	24.03 ± 2.60	0.24
Etiology (%)			1.00
Hepatitis B virus	66 (91.7)	22 (95.7)	
Hepatitis C virus	3 (4.2)	1 (4.3)	
Other	3 (4.2)	0 (0)	
Laboratory results			
Albumin (g/dL)	39.19 ± 4.52	39.40 ± 4.27	0.84
Total bilirubin (umol/L)	17.87 ± 7.48	15.42 ± 5.83	0.16
INR unit	1.04 ± 0.09	1.05 ± 0.12	0.79
AFP (ng/ml)			*<0.001
<20	29	19	
≥20	43	4	
CEA (ng/mL)			0.63
<5	65	20	
≥5	4	2	
CA125 (U/ml)			1.00
<24	55	18	
≥24	14	4	
CA199 (U/ml)			0.90
<25	48	15	
≥25	21	7	
HCC features			
Number of lesions	76	24	
Lesion size(cm)	4.28 ± 3.33	4.39 ± 3.25	0.89
Edmondson-Steiner grade			0.08
1-2	42	8	
3-4	31	14	
MVI			0.90
Positive	22	7	
Negative	50	17	
Ki-67			
<20%	20	12	*0.03
≥20%	56	12	
Liver fibrosis grade			0.79
S1-2	21	8	
S3-4	45	25	
Liver inflammation grade			0.33
A1	16	8	
A2-4	50	15	

HCC, hepatocellular carcinoma; BMI, body mass index; AFP, α-fetoprotein; INR, international normalized ratio of prothrombin time; MVI, microvascular invasion.

Data are mean ± standard deviation unless otherwise indicated. —Other etiological factors include alcoholism, nonalcoholic fatty liver disease, and schistosomiasis. —Missing data for CEA (4 cases), CA125 (4 cases), CA199 (4 cases), Edmondson-Steiner grade (5 cases), MVI (4 cases), liver inflammation grade (6 cases), and liver fibrosis grade (6 cases). *P < 0.05.

### MRI characteristics of participants

**Figure 2:**
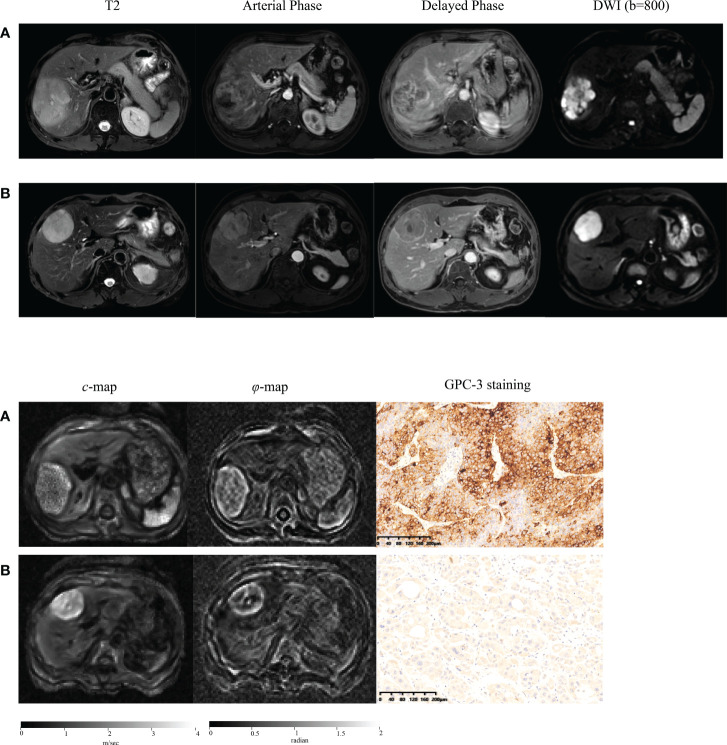
Representative axial T2-weighted images; arterial phase images; delayed phase images; axial diffusion-weighted images (DWI) at *b*-value of 800 sec/mm^2^; axial tomoelastography *c* and *φ* maps, and immunochemistry-stained section images of tumors (magnification, ×20) obtained in a patient with GPC3-positive HCC **(A)** and in a patient with GPC3-negative HCC **(B)**.

Representative axial T2w images, arterial phase images, and delayed phase images for participants with (a) positive and (b) negative GPC3 expression are shown in **Figure 2**. Immunohistochemical analyses of GPC3 were also shown for these two patients where the cytoplasmic/membranous staining of GPC3 was significantly higher in patient with positives GPC3 expression. LI-RADS categories are summarized in **Table 2**. There were no LR1 or LR 2 cases among our participants, and the majority of lesions were categorized as LR-5 (73%). The presence of imaging features such as non-rim APHE (*P* = 0.68), washout (*P* = 0.51), and enhancing capsule (*P* = 0.69) did not differ significantly between the two groups. The distribution of LI-RADS categories was also similar between the two groups (*P* = 0.40).

**Table 2:** Standard MRI features and LI-RADS categories of HCC lesions in the two groups.

	GPC3-positive group (n=76)	GPC3-negative group (n=24)	P-value
Non-rim APHE	62	18	0.68
Washout (not peripheral)	50	14	0.51
Enhancing capsule	44	15	0.69
LI-RADS categories			0.40
LR-M	3	3	
LR-TIV	2	0	
LR-3	3	2	
LR-4	11	2	
LR-5	56	17	

APHE, arterial phase enhancement; LI-RADS, Liver Imaging Reporting & Data System; TIV, Tumor In Vein.

### Mechanical properties of HCC and background liver

**Figure 3:**
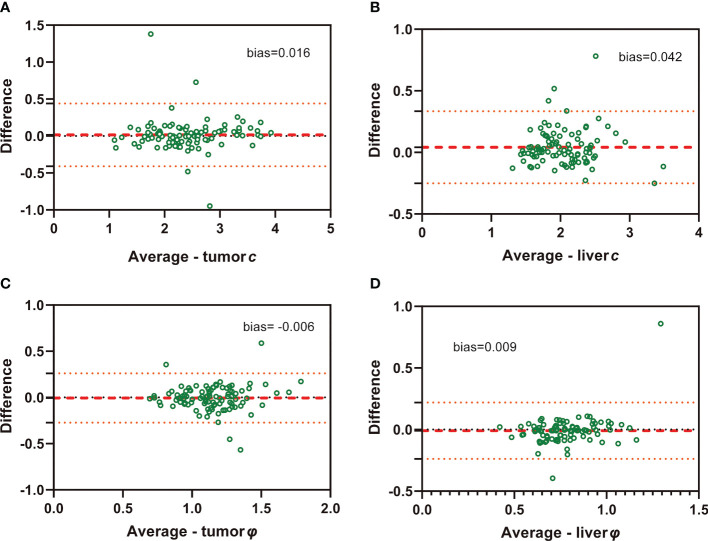
Bland-Altman plots show agreement of c and φ values evaluated by two independent raters in both HCC (**A**: c value of tumor; **C**: φ value of tumor) and background liver (**B**: c value of liver; **D**: φ value of liver)..

ICC of interobserver reliability of mechanical properties based on all participants evaluated by two raters was 0.942 (95% CI: 0.916, 0.961) for tumor *c*, 0.809 (95% CI: 0.728, 0.827) for tumor *φ*, 0.928 (95% CI: 0.889, 0.952) for background liver *c*, and 0.741 (95% CI: 0.635, 0.820) for background liver *φ*, suggesting good concordance and data consistency. Bland-Altman plots are shown in **Figure 3**.

**Figure 4:**
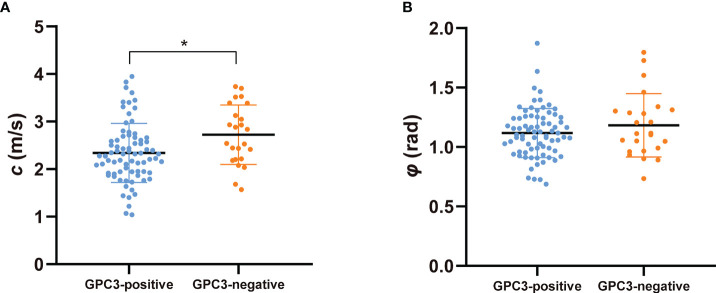
Scatter plots of mechanical parameters **(A)** *c* (stiffness) and **(B)** *φ* (fluidity) comparing GPC3-positive and GPC3-negative groups. *P < 0.05.

**Figure 2** shows tomoelastography *c* and *φ* maps of GPC3-positive and negative participants. It is apparent that the GPC3-postivie HCC is softer (lower *c*-value) than the GPC3-negative tumor. We observed significantly lower *c* values in HCCs of the GPC3-positive group (2.34 ± 0.62 vs 2.72 ± 0.62 m/s, *P* = 0.01) than those of GPC3-negative group. HCC *φ* was not different between these two groups (1.11 ± 0.21 vs 1.18 ± 0.27 rad, *P* = 0.21). In background liver, neither *c* (2.06 ± 0.40 vs 2.08 ± 0.43 m/s, *P* = 0.87) nor *φ* (0.76 ± 0.15 vs 0.81 ± 0.24 rad, *P* = 0.26) was sensitive to GPC3 expression. Group comparisons of the biomechanical properties in both HCCs and background liver are compiled in **Table 3** and plotted in **Figure 4**.

**Table 3:** Group mean and standard deviation of the mechanical parameters *c* (stiffness) and *φ* (fluidity) in the two groups with different GPC3 expression.

Parameters	GPC3-positive group (n=76)	GPC3-negative group (n=24)	P-value
HCC			
*c* (m/s)	2.34 ± 0.62	2.72 ± 0.62	*0.01
*φ* (rad)	1.11 ± 0.21	1.18 ± 0.27	0.21
Background liver			
*c* (m/s)	2.06 ± 0.40	2.08 ± 0.43	0.86
*φ* (rad)	0.76 ± 0.15	0.81 ± 0.24	0.26

### Diagnostic performance of tomoelastography in the prediction of GPC3 expression and comparison with AFP

Univariable and multivariable logistic regression analysis firstly identified tumor *c* (odds ratio [OR], 0.361; 95% CI: 0.158, 0.826; P = 0.02) and serum AFP levels over 20 ng/mL (OR, 8.117; 95% CI: 2.422, 27.199; P = 0.001) as two independent predictors of GPC3-positive HCCs (**Table 4**).

**Table 4:** Univariate and multivariate analyses of variables associated with GPC3-positive HCC.

	Univariable		Multivariable	
	OR (95%CI)	P Value	OR (95%CI)	P Value
Tumor *c* (m/s)	0.484 (0.181, 0.816)	*0.01	0.361 (0.158, 0.826)	*0.02
Tumor *φ* (rad)	0.277 (0.036, 2.106)	0.22		
Liver *c* (m/s)	0.903 (0.283, 2.882)	0.86		
Liver *φ* (rad)	0.236 (0.019, 2.983)	0.26		
Sex				
Male	1			
Female	0.362 (0.076, 1.719)	0.20		
Age	0.982 (0.941, 1.025)	0.42		
BMI	0.914 (0.787, 1.061)	0.24		
Size	0.967 (0.820, 1.139)	0.69		
Albumin (g/dL)	0.989 (0.889, 1.101)	0.84		
Total bilirubin (umol/L)	1.062 (0.977, 1.155)	0.15		
INR unit	0.516 (0.004, 64.255)	0.79		
AFP (ng/mL)		*0.001	8.117 (2.422, 27.199)	*0.001
<20	1			
≥20	7.667 (2.384, 24.650)			
CEA (ng/mL)				
<5	1			
≥5	0.615 (0.105, 3.612)	0.59		
CA125 (U/ml)				
<24	1			
≥24	1.145 (0.334, 3.927)	0.83		
CA199 (U/ml)				
<25	1			
≥25	0.938 (0.334, 2.635)	0.90		
Non-rim APHE				
Absent	1			
Present	2.296 (0.721, 7.315)	0.16		
Nonperipheral washout				
Absent	1			
Present	0.588 (0.226, 1.533)	0.28		
Enhancing capsule				
Absent	1			
Present	0.978 (0.376, 2.542)	0.96		
LI-RADS				
Non-LR-5	1			
LR-5	1.214 (0.437, 3.374)	0.71		

*P < 0.05.

**Figure 5:**
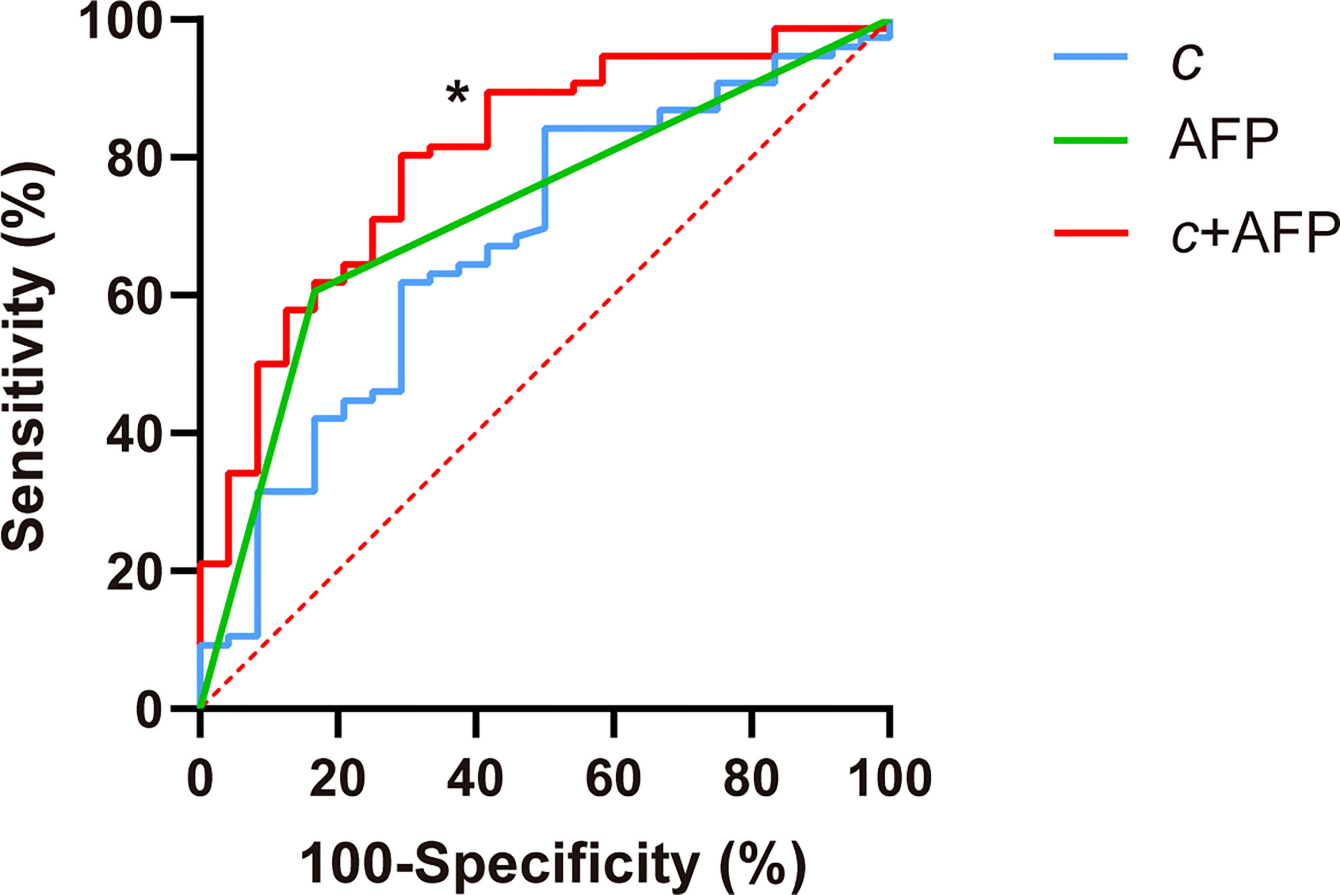
Receiver operating characteristic curves for assessing the diagnostic performance in identifying GPC3-positive HCC using *c* (stiffness), AFP, and both markers combined (*c* + APF). Area-under-the-curve (AUC) values using different biomarkers were compared with respect to that of *c* using the Delong test for *c.* *P < 0.05.

*c* detected GPC3-positive HCCs with an AUC of 0.67 (95% CI: 0.57-0.76, cutoff: 2.8 m/s), which was similar to the diagnostic performance of AFP (AUC: 0.72, 95% CI: 0.62-0.80; cutoff: 20mg/L; *P* = 0.57). Based on AUC analysis, *c* and AFP had high sensitivity (84.2%) and specificity (83.3%), respectively. Therefore, these two parameters were combined for predicting GPC3-positive HCCs. As shown in **Table 5** and **Figure 5**, the joined use of c and AFP yielded a significantly higher AUC of 0.803 (95% CI: 0.711-0.876) compared with using either c (P = 0.024) or APF (P = 0.0065).

**Table 5:** Diagnostic performance of mechanical parameters *c* (stiffness) and serum marker AFP in detecting GPC3-positive HCC.

	Cutoff	AUC	P-value	Sensitivity (%)	Specificity (%)
*c* (m/sec)	2.8	0.674 [0.572-0.764]	…	84.2 (64/76) [74.0-91.6]	50.0 (12/24) [29.1-70.9]
AFP level (≥20mg/L)	20	0.719 [0.621-0.805]	0.57	60.5 (46/76) [48.6-71.6]	83.3 (20/24) [62.6-95.3]
*c* + AFP	…	0.803 [0.711-0.876]	*0.02	80.3 (61/76) [69.5-88.5]	70.8 (17/24) [48.9-87.4]

Unless otherwise specified, data in parentheses are numerators/denominators and data in brackets are 95% CIs. AUC of combined stiffness(c) and AFP level, denoted as c + AFP, was obtained by using probabilities estimated from logistic regression.

## Discussion

With the urgent clinical need for improving HCC immunotherapy, noninvasive detection of GPC3 expression of HCC is of great interest. In this study, we have investigated the macroscopic mechanical manifestation of GPC3 expression using viscoelastic parameters quantified by *in vivo* tomoelastography. A key finding of our study was that GPC3-positive HCC had lower stiffness than GPC3-negative HCC. Diagnostic power in predicting GPC3-positive HCC was highest for the combination of tomoelastography-quantified tumor stiffness and serum marker AFP.

The conventional LI-RADS categories for imaging-based classification of HCC were not sensitive to GPC3 expression in our study population, suggesting that tumor morphology and vascularity are not directly linked to GPC3 upregulation. Tissue stiffness quantified by tomoelastography, on the other hand, was found to be sensitive to GPC3 expression. Our observation that GPC3-positive HCCs were softer than GPC3-negative HCCs seems counterintuitive at first glance, considering that malignant liver lesions usually have higher stiffness than benign tumors (27). However, rather than comparing malignant with benign tumors, we here addressed the influence of a specific protein within groups of malignant HCCs, which both had abnormally high stiffness values compared with surrounding liver tissue and other benign lesions reported in the literature (27). Another study suggests that GPC3-possitive HCCs possess more metastatic potential since GPC3 expression promotes cancer cell proliferation and epithelial-mesenchymal transition (EMT) (10). Several cell biomechanics studies confirm that metastatic cancer cells become soft, which promotes unjamming and facilitates invasion through interfaces and blood vessels (31–33). Recently, EMT has been reported to cause cancer cells to soften and migrate into their matrix environments (34, 35). The biomechanical properties of surrounding tissue can also affect the stiffness of the embedded lesion (33, 36). However, we observed no difference in the biomechanical properties of background livers between the two groups of participants, suggesting that the observed soft signature of GPC3-positive HCCs reflects tumor-intrinsic properties that are the collective behavior of soft and unjammed cancer cells due to GPC3-promted EMT (37).

Tissue fluidity, as quantified by loss angle *φ*, showed no sensitivity to GPC3 expression. Since GPC3 is known to upregulate cell motility, we expected GPC3-rich HCC to behave more fluid-like than GPC3-negative HCC, similar to high-grade tumors in the prostate (24, 38). However, considering that GPC3 is a proteoglycan with negatively charged heparan sulfate chains, its hydrophilic water-binding capacity on HCC cell surfaces could reduce water mobility and turn tissue into a more solid-like state, as known for high-grade glioma in the brain (22). Thus, we hypothesized that the counteracting effects of cell unjamming and water immobilization render *φ* insensitive to GPC3 expression in HCC.

The best diagnostic performance in detecting GPC3-positive HCCs was achieved by combing stiffness and AFP level. The high specificity of AFP level for tumors with GPC3 expression might be related to the shared transcription factors zinc fingers, AFP regulator 2 (Arf2), and homeoboxes 2 (Zfh2) (39–41). As AFP is a serum marker commonly used for clinical HCC screening, it is routinely available. In our study population, the insufficient sensitivity of AFP in detecting GPC3-positive HCC was well compensated for by stiffness, which lacks specificity. Therefore, combining the serum biomarker and the tomoelastography-derived imaging biomarker may be a promising approach for the identification of GPC3-postive HCC. Owing to its noninvasive nature, tomoelastography could also be of value for monitoring and predicting outcome of immunotherapy targeting GPC3.

Our study has limitations. First, it was a single-center study. A multicenter study including other hospitals where tomoelastography is availability is planned. Second, the sample size was relatively small, especially in the GPC3-negative group. However, patient distribution in the GPC3-positive and -negative groups reflects the demographic distribution of GPC3-positive cases in a general HCC population (10). Third, clinical MRI examinations were performed on different scanner systems. However, LI-RADS categories are based on qualitative interpretation of MR images and, according to the guidelines, are not system-dependent (29). Finally, the scope of our study didn’t cover the post-surgical outcome assessment which is of high interest and relevance. This aspect will be incorporated in our future studies to extend the prognostic value of our method.

In summary, reduced stiffness quantified by *in vivo* tomoelastography is a mechanical signature of GPC3-positive HCC. The macroscopic softening observed in GPC3-positive HCCs could be a collective reflection of HCC cell softening as a result of EMT. The combined use of HCC stiffness and AFP level provided high diagnostic accuracy in detecting GPC3 expression and could be considered a viable biomarker for identifying GPC3-positive HCC and predicting therapeutic outcome.

## Data availability statement

The raw data supporting the conclusions of this article will be made available by the authors, without undue reservation.

## Ethics statement

The studies involving human participants were reviewed and approved by Ruijin Hospital Ethics Committee, Shanghai Jiaotong University School of Medicine (No. RJ2018-209). The patients/participants provided their written informed consent to participate in this study.

## Author contributions

Y.W: - Data curation, Formal analysis, Methodology, Visualization, Writing – original draft, Writing – review & editing JG: - Methodology, Resources, Writing – original draft, Writing – review & editing DM: - Data curation, Investigation, Resources JZ: - Data curation, Investigation YY: - Data curation, Investigation, Resources YC: - Project administration, Resources, Supervision HW: - Data curation, Formal analysis, Resources IS: - Resources, Writing – original draft, Writing – review & editing RL: - Conceptualization, Funding acquisition, Methodology, Project administration, Resources, Writing – original draft, Writing – review & editing FY: - Funding acquisition, Supervision, Writing – original draft, Writing – review & editing. All authors contributed to the article and approved the submitted version.

## Funding

RL, YW, JZ, FY are supported by Shanghai Science and Technology Fundation (21TS1400600). IS, JG are supported by the Deutsche Forschungsgemeinschaft (BIOQIC GRK 2260 and SFB1340 Matrix-In-Vision).

## Conflict of interest

The authors declare that the research was conducted in the absence of any commercial or financial relationships that could be construed as a potential conflict of interest.

## Publisher’s note

All claims expressed in this article are solely those of the authors and do not necessarily represent those of their affiliated organizations, or those of the publisher, the editors and the reviewers. Any product that may be evaluated in this article, or claim that may be made by its manufacturer, is not guaranteed or endorsed by the publisher.
