# Supplementary material for: Reduced tumor stiffness quantified by tomoelastography as a predicative marker for glypican-3-positive hepatocellular carcinoma
Source: Front Oncol. 2022 Nov 28;12:962272. doi: 10.3389/fonc.2022.962272 (PMC9744252; doi:10.3389/fonc.2022.962272)
Supplement: Supplementary file 1 [file Table_1.docx]

**Supplemental Table 1**: Multiparametric MRI protocol.

|  | 1.5T Siemens | | | | | |
| --- | --- | --- | --- | --- | --- | --- |
|  | T1w | T2w | | DWI | DCE | |
|  | Axial | Axial | Coronal | Axial | Axial | Coronal |
| TR (msec) | 4.85 | 3000 | 1200 | 6600 | 4.85 | 3.90 |
| TE (msec) | 2.41 | 95 | 91 | 62 | 2.41 | 1.49 |
| Flip angle (degree) | 10 | 140 | 180 | 180 | 10 | 10 |
| Field of view (mm^2^) | 380×309 | 380×380 | 380×380 | 380×306 | 380×3093 | 360×360 |
| Matrix size | 320×240 | 320×320 | 256×256 | 134×134 | 20×240 | 320×234 |
| Section thickness (mm) | 3 | 5 | 6 | 5 | 3 | 3 |

|  | 3.0T Philips | | | | | |
| --- | --- | --- | --- | --- | --- | --- |
|  | T1w | T2w | | DWI | DCE | |
|  | Axial | Axial | Coronal | Axial | Axial | Coronal |
| TR (msec) | 3.6 | 2000 | 1100 | 1520 | 3.7 | 5.2 |
| TE (msec) | 1.31 | 66 | 80 | 70 | 1.32 | 1.07 |
| Flip angle (degree) | 10 | 90 | 90 | 90 | 10 | 10 |
| Field of view (mm^2^) | 400×350 | 360×360 | 450×450 | 327×399 | 400×352 | 380×380 |
| Matrix size | 252×217 | 288×288 | 320×282 | 108×128 | 268×234 | 212×212 |
| Section thickness (mm) | 4 | 5 | 5 | 5 | 4 | 4.4 |

|  | 3.0T United Imaging | | | | | |
| --- | --- | --- | --- | --- | --- | --- |
|  | T1w | T2w | | DWI | DCE | |
|  | Axial | Axial | Coronal | Axial | Axial | Coronal |
| TR (msec) | 120 | 4000 | 1200 | 4258 | 4.15 | 4.31 |
| TE (msec) | 2.28 | 79.92 | 106.04 | 75 | 1.86 | 1.96 |
| Flip angle (degree) | 70 | 90 | 125 | 90 | 10 | 10 |
| Field of view (mm^2^) | 410×280 | 380×360 | 400×380 | 380×300 | 400×300 | 360×350 |
| Matrix size | 288×80 | 320×90 | 256×85 | 128×100 | 256×80 | 256×73 |
| Section thickness(mm) | 6 | 6 | 7 | 6 | 5 | 6 |

Note. —b values of DWI were 0, 50, and 800 sec/mm^2^. TE = echo time, TR = repetition time.
